# Crimean-Congo Hemorrhagic Fever Virus, Mongolia, 2013–2014

**DOI:** 10.3201/eid2412.180175

**Published:** 2018-12

**Authors:** Matthew A. Voorhees, Susana L. Padilla, Dulamjav Jamsransuren, Jeffrey W. Koehler, Korey L. Delp, Dolgorkhand Adiyadorj, Uyanga Baasandagwa, Battsetseg Jigjav, Scott P. Olschner, Timothy D. Minogue, Randal J. Schoepp

**Affiliations:** United States Army Medical Research Institute of Infectious Diseases, Fort Detrick, Maryland, USA (M.A. Voorhees, S.L. Padilla, J.W. Koehler, K.L. Delp, S.P. Olschner, T.D. Minogue, R.J. Schoepp);; Ministry of Health National Center for Zoonotic Diseases, Ulaanbaatar, Mongolia (D. Jamsransuren, D. Adiyadorj, U. Baasandagwa, B. Jigjav)

**Keywords:** Crimean-Congo hemorrhagic fever, CCHF, CCHFV, Mongolia, serosurvey, ticks, tickborne disease, vector-borne infections, viruses, zoonoses, *Hyalomma asiaticum*, *Dermacentor nuttalli*, S segment, M segment

## Abstract

During 2013–2014, we collected 1,926 serum samples from humans and 4,583 ticks (*Hyalomma asiaticum* or *Dermacentor nuttalli*) in select regions of Mongolia to determine the risk for Crimean-Congo hemorrhagic fever virus (CCHFV) infection among humans in this country. Testing of human serum samples by ELISA demonstrated an overall CCHFV antibody prevalence of 1.4%; Bayankhongor Province had the highest prevalence, 2.63%. We pooled and analyzed tick specimens by real-time reverse transcription PCR; 1 CCHFV-positive *H. asiaticum* tick pool from Ömnögovi was identified. In phylogenetic analyses, the virus’s partial small segment clustered with CCHFV isolates from Central Asia, and the complete medium segment grouped with CCHFV isolates from Africa, Asia, and the Middle East. This study confirms CCHFV endemicity in Mongolia and provides information on risk for CCHFV infection. Further research is needed to better define the risk for CCHFV disease to improve risk mitigation, diagnostics, and surveillance.

Crimean-Congo hemorrhagic fever (CCHF) is a tickborne disease of humans that is prevalent over a wide geographic area, spanning from western China to southern Asia, from the Middle East to southeastern Europe, and over most of Africa (*1*–*3*). The causative agent is Crimean-Congo hemorrhagic fever virus (CCHFV), a member of the *Bunyaviridae* family and *Orthonairovirus* genus. The enveloped virus has a negative-sense RNA genome composed of 3 ambisense segments: small (S), medium (M), and large (L). The S segment encodes the nucleocapsid protein and the L segment the viral polymerase. The M segment encodes a glycoprotein precursor that is processed into 2 glycoproteins by the host cell. Genetic drift and segment reassortment of the CCHFV genome results in a high degree of phylogeographic diversity, with 5–7 designated genotypes (*2*,*4*).

CCHFV is maintained in the environment in a cycle between tick vectors, primarily *Hyalomma* ticks, and wild and domesticated animals. Birds generally appear to be refractory to CCHFV infection, although some avian species demonstrate infectivity (*5*). Widespread dispersion is most likely mediated by infected ticks carried by migrating birds or moving livestock (*1*,*6*–*8*). Viremia in mammals is transient, but ticks remain infected throughout their lives (*9*). The human species is considered a dead-end host because virus transmission from infected humans to uninfected feeding ticks has not been reported. Humans can become infected when fed on by infected ticks, exposed to infectious bodily fluids during animal slaughter, or caring for infected patients (*1*,*10*). Infection can result in an unapparent or mild disease of fever, chills, nausea, vomiting, headache, and body pains. In severe cases, hemorrhagic disease is characterized by petechial rashes of the skin, bleeding from mucosal membranes and muscles, organ failure, and cerebral hemorrhage. Mortality rates have been estimated to be 5%–30% but have reached as high as 80% in some outbreaks (*10*). One report describes CCHF survivors as having lasting effects, including prolonged vision and memory problems (*11*).

CCHFV prevalence in Mongolia is generally unknown. No published studies provide evidence of human infection or of virus circulating in tick vectors. A previous study in the Mongolia aimag (i.e., province) of Arkhangai demonstrated serologic evidence of virus infection in 1.8% of wildlife species tested (*12*), and a large study involving >2,000 samples collected from sheep demonstrated a CCHFV seroprevalence of 27.1% among sheep from multiple aimags across Mongolia (*13*). Furthermore, numerous CCHFV strains have been identified in Xinjiang Province, a region of China bordering Mongolia (*14*–*16*). Serologic evidence of CCHFV infections in wildlife and livestock suggests the virus circulates in Mongolia and that CCHFV infections are possible for persons exposed to biting ticks or infectious tissues or fluids. In this study, we sought to better characterize the risk for CCHFV infection among the population of Mongolia by determining antibody prevalence in humans associated with livestock and by surveying ticks collected from the environment and livestock for evidence of CCHFV infection.

## Materials and Methods

### Human Samples

**Figure 1:**
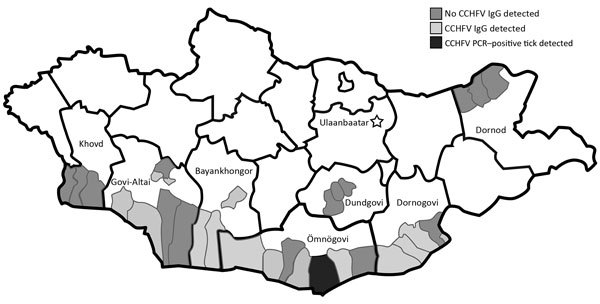
Geographic distribution of CCHFV-positive serum samples and tick and CCHFV-negative serum samples, Mongolia, 2013–2014. CCHFV, Crimean-Congo hemorrhagic fever virus.

During 2013–2014, we collected serum samples (n = 1,926) from persons in regions corresponding to the estimated geographic distribution of *Hyalomma asiaticum* ticks (*17*); we collected most samples in 2013. We collected samples from Dornod Aimag in eastern Mongolia; Dornogovi Aimag, Dundgovi Aimag, Ömnögovi Aimag, Bayankhongor Aimag, and Govi-Altai Aimag in southern Mongolia (the Gobi Desert landscapes); and Khovd Aimag in western Mongolia (Table 1; Figure 1). We collected blood from nomadic herders and local residents of both sexes from the selected regions by using standard blood sampling techniques. We centrifuged and stored serum samples at −20°C until transporting them to the National Center for Zoonotic Diseases (Ulaanbaatar, Mongolia), where samples were stored at −70°C. We collected all human samples in accordance with Mongolia Ministry of Health−approved protocols and tested all human samples at the US Army Medical Research Institute of Infectious Diseases (USAMRIID) under approved human use protocol FY11–14.

**Table 1:** Prevalence of CCHFV IgG among healthy persons as determined by ELISA, by province and district, Mongolia, 2013–2014*

Province, district	No. positive/tested (%)		
	2013	2014	Total
Khovd	0/229 (0)	NA	0/229 (0)
Üyench	0/79 (0)		0/79 (0)
Bulgan	0/72 (0)		0/72 (0)
Altai	0/78 (0)		0/78 (0)
Govi-Altai	3/314 (0.96)	1/21 (4.76)	4/335 (1.19)
Bugat	2/84 (2.38)		2/84 (2.38)
Altai	1/84 (1.19)		1/84 (1.19)
Tsogt	0/74 (0)		0/74 (0)
Erdene	0/72 (0)		0/72 (0)
Taishir		0/5 (0)	0/5 (0)
Yesönbulag		1/16 (6.25)	1/16 (6.25)
Bayankhongor	4/146 (2.74)	1/44 (2.27)	5/190 (2.63)
Bayan-Öndör	2/74 (2.70)	0/1 (0)	2/75 (2.67)
Shinejinst	2/72 (2.78)		2/72 (2.78)
Jinst		1/43 (2.33)	1/43 (2.33)
Ömnögovi	7/525 (1.33)	1/40 (2.50)	8/565 (1.42)
Gurvan-tes	3/79 (3.80)	0/10 (0)	3/89 (3.37)
Noyon	1/71 (1.41)	1/10 (10.00)	2/81 (2.47)
Bayandalai	0/74 (0)	0/10 (0)	0/84 (0)
Khürmen	1/75 (1.33)	0/10 (0)	1/85 (1.18)
Nomgon	1/75 (1.33)		1/75 (1.33)
Bayan-Ovoo	1/75 (1.33)		1/75 (1.33)
Khanbogd	0/76 (0)		0/76 (0)
Dornogovi	6/410 (1.46)	4/72 (5.56)	10/482 (2.07)
Khatanbulag	2/93 (2.15)	2/17 (11.76)	4/110 (3.63)
Khövsgöl	3/91 (3.30)	1/14 (7.14)	4/105 (3.81)
Ulaanbadrakh	0/60 (0)	1/16 (6.25)	1/76 (1.31)
Erdene	0/80 (0)	0/14 (0)	0/94 (0)
Zamyn-Üüd	1/86 (1.16)	0/11 (0)	1/97 (1.03)
Dundgovi	NA	0/76 (0)	0/76 (0)
Khuld		0/33 (0)	0/33 (0)
Luus		0/39 (0)	0/39 (0)
Saintsagaan		0/4 (0)	0/4 (0)
Dornod	NA	0/49 (0)	0/49 (0)
Bayan-Uul		0/14 (0)	0/14 (0)
Bayandun		0/15 (0)	0/15 (0)
Dashbalbar		0/20 (0)	0/20 (0)
Total	20/1,624 (1.23)	7/302 (2.32)	27/1,926 (1.40)

*CCHFV, Crimean-Congo hemorrhagic fever virus; NA, none available.

### IgG ELISA

We detected human CCHFV IgG by sandwich ELISA. We coated Immulon 2 HB plates (ThermoFisher Scientific, Waltham, MA, USA) with anti-CCHFV hyperimmune mouse ascitic fluid (1:1,000) in phosphate-buffered saline and 0.01% thimerosal. After incubating plates overnight at 4°C and washing 3 times with wash buffer (phosphate-buffered saline with 0.1% tween 20 and 0.01% thimerosal), we added 100 µL of inactivated CCHFV IbAr10200 supernatant (IgG capture antigen) or noninfected Vero E6 cell supernatant (negative control) diluted (1:20) in milk buffer (wash buffer plus 5% skim milk) to each well. We incubated plates for 60 min at 37°C, washed 3 times with wash buffer, and added serum samples diluted 1:100 in milk buffer to test wells and negative control wells. We incubated plates for 60 min at 37°C, washed 3 times with wash buffer, and added 100 µL of the detector antibody (horseradish peroxidase–labeled anti-human IgG F_c_; Accurate Chemical and Scientific, Westbury, NY, USA) diluted 1:8,000 in milk buffer to all wells. We incubated plates for 60 min at 37°C, washed 3 times with wash buffer, and developed using 100 µL of substrate 2, 2’-azino-di-(3-ethylbenzothiazoline-6-sulfonate) with a 30-min incubation at 37°C. We read plates using a TECAN Infinite M200 Pro (Tecan Group Ltd., Männedorf, Switzerland) plate reader at 405 nm and determined the optical density (OD) for each well. We calculated the adjusted OD for each sample by subtracting the average OD value of the Vero cell supernatant control wells from the average OD value of the IgG capture antigen wells. The positive-negative cutoff of each assay was the mean OD value plus 3 SDs of the 4 negative control samples (typically 0.2).

### Plaque Reduction Neutralization Test

For the plaque reduction neutralization test (PRNT), we diluted all test samples 1:10 in minimum essential media (MEM) with 5% heat-inactivated Hyclone Fetal Bovine Serum (FBS) (GE Healthcare Life Sciences, Pittsburgh, PA, USA) and then placed samples in a 56°C water bath for 30 min. For test and positive control serum samples, we 2-fold serially diluted to 1:320 in MEM with 5% FBS. For negative control serum samples, we 2-fold serially diluted to 1:40 in the same buffer. We diluted CCHFV IbAr10200 in MEM with 5% FBS to a concentration of 1,000 plaque-forming units/mL.

We mixed positive control, negative control, and test serum samples with virus 1:1 in microtiter tubes, covered tubes with parafilm, and then placed them at 37°C for 1 h. We incubated 200 µL of serum-virus mixture in duplicate wells of 6-well plates with SW-13 cells grown to a minimum of 85% confluence. We incubated plates at 37°C with 5% CO_2_ for 1 h with gentle rocking every 15 min. After incubation, we added 2 mL of 0.5% agar overlay (2× basal medium Eagle with Earle salts with 10% FBS, 1% penicillin-streptomycin, 1% L-glutamine, and 1% nonessential amino acids) to each well and incubated at 37°C for ≈36 h. Then, we added a second 0.5% agar overlay containing 5% neutral red to each well, incubated at 37°C for 4–5 h, and counted plaques. We reported the reciprocal of the highest serum dilutions reducing 50% (PRNT_50_) and 80% (PRNT_80_) of the plaque assay dose as the titer and calculated the probit titer.

### Tick Collections and Processing

We collected ticks from livestock and questing ticks from the environment in the same regions of Mongolia where we collected human serum samples. During the spring and early summer, we collected adult, unfed ticks by the drag cloth method (dragging a cotton cloth on a dowel) and by removing ticks from livestock with forceps. We placed each collection in a centrifuge tube with a moist cotton. In the field, we stored tick collections in portable thermal coolers at 4°C. We sent ticks to the National Center for Zoonotic Diseases, where samples were stored at −20°C until species identification was completed. Afterward, we transferred ticks to −70°C storage until shipment to USAMRIID for processing. We collected 4,583 ticks for processing, of which 1,772 were *H. asiaticum* and 2,811 were *Dermacentor nuttalli*.

We sorted field-collected unfed specimens by sex and species and placed ticks in pairs into single polycarbonate vials containing prepared media and 3/8-inch steel balls. We homogenized ticks using the 1600 MiniG Automated Tissue Homogenizer and Cell Lyser (SPEX SamplePrep, Metuchen, NJ, USA), combined portions of homogenized subpools into larger pools (maximum 8 ticks/pool), and treated them with TRIzol LS (ThermoFisher Scientific) according to the manufacturer’s instructions. We purified total nucleic acid using the KingFisher Flex Purification System (ThermoFisher Scientific) and the MagMax 96 for MicroArrays Total RNA Isolation Kit (ThermoFisher Scientific) according to the manufacturer’s recommendations. We stored all tick pools and processed nucleic acids at −70°C until needed for testing.

### Real-Time Reverse Transcription PCR

We tested total nucleic acid extracted from tick pools for the CCHFV S segment using a real-time reverse transcription PCR (RT-PCR) assay optimized for CCHFV sequence diversity (*18*,*19*). We performed this assay in triplicate in wells of 384-well plates using the SuperScript III One-Step RT-PCR Kit (Invitrogen, Carlsbad, CA, USA) with 2.5 µL of sample in 10-µL reactions run on the LightCycler 480 (Roche Applied Science, Penzberg, Germany). Cycling conditions were 50°C for 15 min; 95°C for 5 min; and 45 cycles of 94°C for 1 s, 55°C for 20 s, and 68°C for 5 s. Fluorescence was measured after each extension step. We considered a sample negative if the quantification cycle was >40 cycles.

### Nested RT-PCR

To confirm real-time RT-PCR results, we performed nested RT-PCR targeting the CCHFV S segment as previously described (*20*). In brief, we used 15 µL of extracted tick homogenate and SuperScript III One-Step RT-PCR System with Platinum *Taq* High Fidelity DNA Polymerase (Invitrogen) in a 50-µL reaction with outer primers CCHF-F2 (5′-TGGACACCTTCACAAACTC-3′), CCHF-F2C (5′-TGGATACTTTCACAAACTC-3′), and CCHF-R3 (5′-GACAAATTCCCTGCACCA-3′). Cycling conditions were 42°C for 30 min; 94°C for 5 min; 5 cycles of 94°C for 30 s, 37°C for 30 s, and 72°C for 30 s; 72°C for 2 min; 30 cycles of 94°C for 30 s, 52°C for 30 s, 72°C for 30 s; and a final cycle of 72°C for 5 min. We assessed for the presence of amplicons (536 bp) by performing electrophoresis on 2% agarose gels.

We performed nested PCR with 1 µL of amplicon as a template in a 50-µL reaction with inner primers CCHF-F3 (5′-GAATGTGCATGGGTTAGCTC-3′), CCHF-F3C (5′-GAGTGTGCCTGGGTTAGCTC-3′), CCHF-R2a (5′-GACATCACAATTTCACCAGG-3′), and CCHF-R2b (5′-GACATTACAATTTCGCCAGG-3′). Cycling conditions were 94°C for 5 min; 30 cycles of 94°C for 30 s, 52°C for 30 s, 72°C for 30 s; and 72°C for 5 min. We assessed for the presence of amplicons (260 bp) by performing electrophoresis on 2% agarose gels.

### CCHFV Sequencing and Analysis

We performed a second RT-PCR with S segment outer primers with the only tick pool that was positive by real-time and nested RT-PCR (159A). We then used 5 µL of the reaction for Sanger sequencing. We ran the amplicon (536 bp, near 5′ end of S segment) through a 2% agarose gel, cut the band out, and purified the amplicon using the QIAquick Gel Extraction Kit (QIAGEN, Dusseldorf, Germany). We created sequencing libraries using the BigDye Terminator v3.1 Sequencing Kit (Applied Biosystems, Foster City, CA, USA) with 10 µL of the purified amplicon in a 20-µL reaction. Cycling conditions were 96°C for 1 min and 25 cycles of 96°C for 10 s, 50°C for 5 s, and 60°C for 4 min. We sequenced libraries using the 3500xL Genetic Analyzer (Applied Biosystems).

We attempted to amplify CCHFV segments L, M, and S from tick pool 159A using segment-specific primers modified for Nextera-based sequencing (Table 2) (*21*,*22*); however, only the M segment amplified. In brief, we used 5 µL of extracted nucleic acid from tick homogenate to reverse transcribe the genome and amplify the cDNA of the M segment using the SuperScript III One-Step RT-PCR System with Platinum *Taq* High Fidelity DNA Polymerase (Invitrogen). Cycling conditions were 52.5°C for 30 min; 94°C for 2 min; 40 cycles of 94°C for 15 s, 50.5°C for 30 s, and 68°C for 1 min/kb; and 68°C for 5 min. We ran the amplified M segment through an agarose gel and purified the band using the QIAquick Gel Extraction Kit (QIAGEN).

**Table 2:** Primer sequences used for Nextera-based sequencing of S, M, and L segments of CCHFV from tick pool 159A, Mongolia, 2013–2014*

Primer name	Sequence, 5′ → 3′†
CCHFV-M F-next	TCGTCGGCAGCGTCAGATGTGTATAAGAGACAGtctcaaagaaatacttgc
CCHFV-M R-next	GTCTCGTGGGCTCGGAGATGTGTATAAGAGACAGtctcaaagatatagtggc
CCHFV-S F-next	TCGTCGGCAGCGTCAGATGTGTATAAGAGACAGtctcaagaaacacgtgccgc
CCHFV-S R-next	GTCTCGTGGGCTCGGAGATGTGTATAAGAGACAGtctcaaagatatcgttgccgc
CCHF-L 1F-next	TCGTCGGCAGCGTCAGATGTGTATAAGAGACAGtctcaaagatatcaatcccccc
CCHF-L 1R-next	GTCTCGTGGGCTCGGAGATGTGTATAAGAGACAGttggcactatctttcatttga
CCHF-L 2F-next	TCGTCGGCAGCGTCAGATGTGTATAAGAGACAGgaagagctatatgacataaggc
CCHF-L 2R-next	GTCTCGTGGGCTCGGAGATGTGTATAAGAGACAGtctcaaagaaatcgttccccccac

*CCHF, Crimean-Congo hemorrhagic fever; CCHFV, Crimean-Congo hemorrhagic fever virus; L, large; M, medium; S, small. †The virus-specific sequences are lowercase, and the Nextera-specific sequences are capitalized.

We generated next-generation sequencing libraries using the Nextera XT DNA Library Preparation Kit (Illumina, San Diego, CA, USA) according to the manufacturer’s instructions and sequenced using the 500-cycle MiSeq Reagent Kit v2 (Illumina). We analyzed sequencing reads with the CLC Genomics Workbench (QIAGEN), filtered and trimmed them for quality, and assembled the complete M segment de novo. We generated the final consensus sequence by remapping the trimmed reads to the de novo consensus sequence as previously described (*22*).

We identified the full-length M segment sequence and partial S segment sequence from tick pool 159A by BLAST analysis (https://blast.ncbi.nlm.nih.gov/Blast.cgi). We aligned the segments from tick pool 159A with the available full-length M and near–full-length S segments in GenBank and generated a neighbor-joining phylogenetic tree (Jukes-Cantor model) with 1,000 bootstrap replicates.

### Virus Isolation

To propagate virus in the RT-PCR–positive tick pool, we thawed 60 µL of tick homogenates and mixed with 6 mL of freshly prepared MEM with 10% FBS, 1% nonessential amino acids, 1% L-glutamine, 1% penicillin-streptomycin, and 0.4% fungizone. We inoculated confluent monolayers of SW-13 cells in 2 T75 flasks with 2.5 mL of the virus mixture and incubated at 37°C for 1 h; then, we added an additional 30 mL of MEM. We incubated flasks at 37°C with 5% CO_2_ for 1 wk or until cytopathic effects (CPE) were evident. To test for unapparent infections (i.e., infections not resulting in CPE), each inoculated culture was tested by real-time RT-PCR as previously described.

## Results

### CCHFV Serologic Testing in Humans

To assess the overall risk for CCHFV infection in Mongolia, we tested 1,926 serum samples collected from nomadic herders and local residents in select regions of the country by ELISA. In total, 27 samples tested positive for CCHFV IgG; the overall prevalence rate was 1.4% (Table 1). Bayankhongor Aimag had the highest prevalence (2.63%); other aimags with CCHFV IgG–positive populations had prevalences of 2.07% (Dornogovi), 1.42% (Ömnögovi), and 1.19% (Govi-Altai). The remaining 3 aimags tested (Khovd, Dundgovi, and Dornod) showed no evidence of CCHFV IgG.

We evaluated 12 positive and 7 randomly selected negative samples for their ability to neutralize CCHFV strain IbAr10200 (Table 3). In total, 8 (67%) of 12 ELISA-positive samples and 2 (29%) of 7 ELISA-negative samples neutralized CCHFV with PRNT_50_ probit titers; 5 samples with PRNT_50_ probit titers (3 ELISA-positive and 2 ELISA-negative) had PRNT_80_ probit titers. Neutralization ability did not correlate with ELISA OD values.

**Table 3:** CCHFV neutralization capacity of human serum samples with and without evidence of CCHFV IgG by ELISA, Mongolia, 2013–2014*

Sample no.	ELISA OD	PRNT_50_ probit titer†	PRNT_80_ probit titer†
376	0	<10	<10
674	0	<10	<10
272	0	89	27
822	0	<10	<10
408	0	19	11
868	0.01	<10	<10
85	0.02	<10	<10
867	0.21	<10	<10
862	0.31	26	<10
297	0.38	29	10
41	0.40	33	<10
667	0.42	15	<10
823	0.43	<10	<10
115	0.64	<10	<10
101	0.87	<10	<10
131	0.97	10	<10
870	1.18	127	45
81	1.18	40	20
97	1.28	20	<10

*Samples positive for CCHFV IgG by ELISA were those with ODs >0.20. OD, optical density; PRNT_50_, plaque assay neutralization test with 50% of the plaque assay dose reduced; PRNT_80_, plaque assay neutralization test with 80% of the plaque assay dose reduced. †The titer reported is the inverse of the highest serum sample dilution that neutralized the appropriate percentage of virus.

### Molecular Testing for CCHFV in Ticks

We collected questing ticks and ticks from livestock and identified and pooled them on the basis of tick species, sex, and geographic location; then, we screened tick pools by real-time RT-PCR using a broad CCHFV assay (*19*). Of the 893 tick pools tested, 1 pool (159A) tested positive by real-time RT-PCR and was confirmed CCHFV positive by nested RT-PCR; results for 6 other pools were indeterminate. Tick pool 159A, which contained 7 *H. asiaticum* ticks, was collected in the soum (i.e., district) Nomgon in Ömnögovi Aimag.

### Characterization of CCHFV-Positive Pool 159A

**Figure 2:**
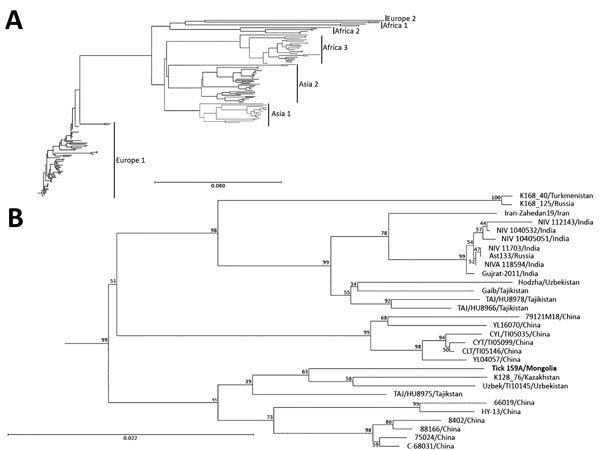
Phylogenetic characterization of partial small (S) segment sequence of Crimean-Congo hemorrhagic fever virus (CCHFV) isolate from tick pool 159A, Mongolia, 2013–2014. Near full–length CCHFV S segments from GenBank were aligned with the S segment sequence from tick pool 159A and a phylogenetic tree was generated. A) Genetic clusters are displayed as previously described (*23*). B) Detailed view of phylogenetic tree of Asia 2 lineage. S segment of the CCHFV isolate from this study (tick 159A/Mongolia; bold) clusters in the Asia 2 lineage. Scale bars indicate nucleotide substitutions per site.

**Figure 3:**
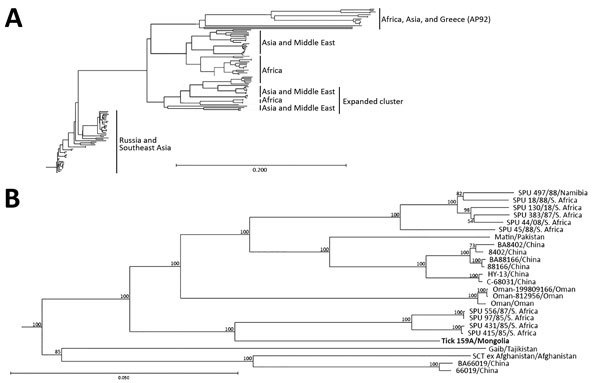
Phylogenetic characterization of full-length medium (M) segment sequence of Crimean-Congo hemorrhagic fever virus (CCHFV) isolate from tick pool 159A, Mongolia, 2013–2014. Full-length and near full–length CCHFV M segments from GenBank were aligned with the M segment sequence of tick pool 159A and a phylogenetic tree was generated. A) M segment lineages weakly cluster by geographic location. B) Detailed view of phylogenetic tree cluster including the isolate from this study (tick 159A/Mongolia; bold). Tick 159A/Mongolia groups with CCHFV isolates from Asia, the Middle East, and Africa. Scale bars indicate nucleotide substitutions per site.

To genetically characterize the CCHFV detected in tick pool 159A, we amplified and sequenced the full-length M segment and partial S segment. BLAST analysis of the nucleocapsid coding region located within the partial S segment identified K128_76 (GenBank no. KX013455.1) from Kazakhstan as the CCHFV isolate having the greatest percent identity at 98%, and IbAr10200, the prototypical CCHFV isolate, had a percent identity of 87%. BLAST analysis of the M segment identified SPU 97/85 (GenBank accession no. DQ211633.1) from South Africa as the CCHFV isolate having the highest percent identity at 91%, with IbAr10200 having a percent identity of 81%. Phylogenetic analysis indicated that the partial S segment sequence clusters within the Asia 2 lineage (Figure 2), which contains virus strains from China and Russia (*23*). CCHFV M segments in general do not cluster well by geographic location on phylogenetic analyses; the M segment sequence tick 159A/Mongolia clustered with CCHFV strains from Asia, Africa, and the Middle East (Figure 3).

### Virus Isolation

Attempts to isolate CCHFV from putative PCR-positive tick pools in SW-13 culture cells were made from multiple tick homogenate subpools (N = 18), containing 2 ticks per subpool. None of the inoculated cultures showed evidence of virus growth by visible CPE or genomic signature by real-time RT-PCR in either the first or second passages.

## Discussion

To better determine the risk for CCHFV infection among humans in Mongolia, we conducted an assessment that included a CCHFV IgG serosurvey and screening for CCHFV RNA in ticks collected in parallel with the serosurvey. Overall, this study provides evidence that CCHFV is circulating in *H. asiaticum* ticks and humans in Mongolia, provides sequence data on a virus circulating in the region, and extends the geographic distribution of CCHFV north to Mongolia.

Previous studies conducted in Mongolia revealed serologic evidence of CCHFV in wildlife species and livestock. Chumikhin et al. found serologic evidence for CCHFV infection in small mammals, such as the Tolai hare, Mongolian pika, and long-tailed ground squirrel (*12*,*13*). Livestock, such as cattle, sheep, goats, and camels, are amplifying hosts of the virus, and CCHFV infections in livestock correlate with the risk for infection of humans (*24*). Morikawa investigated CCHFV IgG seroprevalence in sheep in the southern aimags of Mongolia and found prevalences of 7%–28% (*13*). Evidence of CCHFV in wildlife and livestock in Mongolia suggests the possibility of virus circulation and human disease, but the actual occurrence was unclear.

We found an overall CCHFV IgG seroprevalence of 1.4% in Mongolia; Bayankhongor Aimag had the highest rate, 2.63%. Each soum tested within Bayankhongor had IgG prevalences >2%, suggesting a persistent source of CCHFV infection is likely present in these soums. Bayankhongor is located in the southwestern part of the country and has an economy based on livestock, meat, and wool production. The greater interaction with animals among persons of this aimag could partially explain the higher antibody prevalence in the population. Dornogovi Aimag had the next highest antibody prevalence (2.07%). Dornogovi is in the southeastern part of Mongolia and lies in the eastern part of the Gobi Desert. Raising livestock previously was the main economic driver of this aimag, but this activity has been replaced by mineral exploration. Soums Khatanbulag and Khövsgöl within Dornogovi had some of the highest CCHFV antibody prevalences of those sampled.

Ömnögovi Aimag had an antibody prevalence of 1.42%, despite having the greatest number of samples tested and being the only aimag having a soum with a CCHFV-positive tick pool sample. Ömnögovi, the largest aimag in Mongolia, is located in the southern part of the country and southern part of the Gobi Desert. Ömnögovi is rich in mineral deposits and depends heavily on mining, and agriculture is of minor importance. The lowest antibody prevalence, 1.19%, occurred in the Govi-Altai Aimag, where the Gobi Desert, steppe grasslands, and mountains of western Mongolia converge. No CCHFV IgG were found in samples tested from the other 3 aimags, Khovd, Dundgovi, and Dornod. Dornod Aimag is outside the *Hyalomma* tick range, and thus, we did not expect to find evidence of CCHFV infection in this area.

Antibody neutralization tests were used to confirm the IgG ELISA data because antibody-mediated virus neutralization suggests specificity to the tested virus. We found that 67% of the ELISA-positive samples tested neutralized CCHFV IbAr10200. Because we used an isolate from West Africa in our PRNT, our results are probably an underestimate of the true percentage of samples specific for CCHFV. Nucleic acid differences could account for the reduced neutralization. On the other hand, ELISA-positive, PRNT-negative samples could indicate exposure to related viruses, such as Dugbe virus. Two of 7 randomly selected ELISA-negative samples neutralized CCHFV to some extent, which could have been the result of nonspecific binding or low IgG concentrations below the level of detection of our ELISA.

As further supporting evidence suggesting CCHFV circulation in Mongolia, we identified 1 tick pool positive for CCHFV genetic material, which enabled us to sequence the S and M segments of the isolate and perform phylogenetic analyses. The S segment, which generally clusters on the basis of geographic location in phylogenetic analyses, was similar to other sequenced CCHFV isolates found in countries of Central and East Asia, including Kazakhstan, Uzbekistan, Tajikistan, and China, corresponding to the Asia 2 CCHFV lineage or lineage IV (*21*,*23*). The M segment, which has a higher mutation rate than the S segment, had the greatest homology to isolates from South Africa, the Middle East, and Asia, corresponding to the M segment lineage IV (*21*,*23*).

Overall, our study demonstrated the presence of CCHFV in Mongolia, suggesting human infections are occurring in the area. Our findings are not surprising, considering that others have shown by serology and tick testing the presence of CCHFV in the region (*15*,*25*). Because of Mongolia’s size and diversity, additional studies are required to better characterize the virus and the disease severity it causes. Taken together, these data suggest CCHFV is likely endemic in Mongolia. Further investigations are needed to better define the risk for human infection and the genomic diversity of CCHFV in the region to improve risk mitigation, diagnostics, and surveillance.
